# An optimal model of long-term post-stroke care

**DOI:** 10.3389/fneur.2023.1129516

**Published:** 2023-03-23

**Authors:** Iwona Sarzyńska-Długosz

**Affiliations:** Second Department of Neurology, Institute of Psychiatry and Neurology, Warsaw, Poland

**Keywords:** stroke, standard of care, rehabilitation, spasticity, complication

## Abstract

Stroke is a major healthcare challenge that is increasing worldwide. The burden of stroke is significant for the affected individuals as well as for the general population; high-quality care is needed to reduce its negative impacts. This article synthesized information from systematic reviews, guidelines, and primary literature on stroke care and post-stroke rehabilitation and proposes an optimal strategy for long-term post-stroke care. It also highlights the unmet needs of patients who experienced a stroke in terms of early diagnosis of complications and adequate, comprehensive therapy.

## Introduction

Stroke is one of the greatest public healthcare challenges for the global population. In 2019, there were 12.2 million incident cases and 101 million prevalent cases of stroke worldwide, representing increases of 70 and 85%, respectively, from 1990 (1). The lifetime risk of having a stroke has increased by 50% over the past 17 years, and 1 in 4 people will have a stroke in their lifetime (2). Stroke was also the second-leading cause of death in 2019, with 6.55 million deaths (11.6% of the total), which increased by 43% from 1990 to 2019 (1). Moreover, mathematical models have predicted a 36% increase in the number of stroke events in the European Union (EU) combined with Iceland, Norway, and Switzerland between 2000 and 2025 (3, 4). The disease burden of stroke is accompanied by a substantial economic burden: the total (direct and indirect) costs of stroke were estimated to be $40.1 billion annually in the United States (US) (5) and €45 billion in the EU (3).

Mortality rates alone do not provide the full picture of stroke burden. Stroke survivors are at a high risk of having a stroke in the future; a meta-analysis of 13 studies based on stroke registries estimated the cumulative risk of stroke recurrence as 3.1% in 30 days, 11.1% in 1 year, 26.4% in 5 years, and 39.2% in 10 years (6). Stroke was the fifth leading cause of disability-adjusted life years (DALYs) in 1990 but was the third leading cause by 2010 (7); from 1990 to 2019, DALYs due to stroke increased by 32.0%, accounting for 143 million DALYs in 2019, with 3% of men and 2% of women in the US experiencing disability due to stroke (8). Stroke not only affects the patients but also has a prolonged physical, emotional, and financial impact on their family and friends; up to 48% of caregivers of patients who experienced stroke report health problems and two-thirds have experienced a decline in their social activities (9, 10). As the number of stroke survivors is predicted to increase from 3,718,785 in 2015 to 4,631,050 in 2035 (3), there is an urgent need for improvements in every aspect of stroke care.

**Figure 1:**
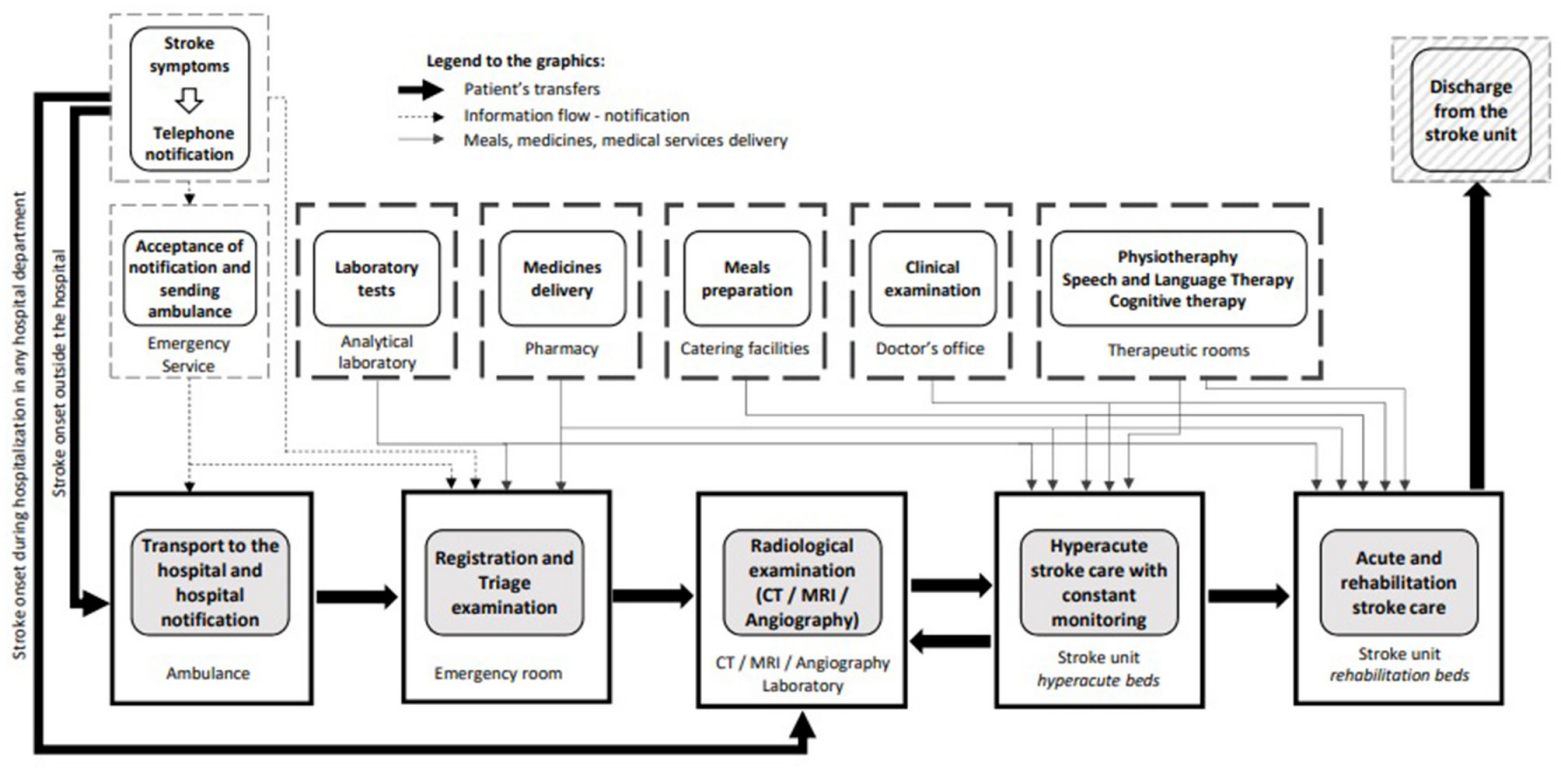
In-hospital acute stroke care pathway (unpublished - drawn by Jarosław Gębski, Polish Society of Health Economics, Warsaw, Poland).

The quality of healthcare is defined as “the degree to which health services for individuals and populations increase the likelihood of desired health outcomes and are consistent with current professional knowledge” (11). Stroke prognosis largely depends on acute-phase care: patients with suspected stroke should be admitted to the hospital as quickly as possible and assessed and treated within a few hours to improve outcomes. The in-hospital acute stroke care pathway is well-established (Figure 1): patients who experienced an acute stroke are transferred directly to stroke units from the emergency department and remain there for the duration of the inpatient stay (12). Stroke units provide multidisciplinary care and rehabilitation by staff specialized in stroke care. The effectiveness of high-quality stroke units is paramount: regardless of age, sex, disability, or stroke type, patients who receive organized in-patient care in a stroke unit have higher survival rates and achieve independence more rapidly, and they will be sooner able to return to their own home (12). At present, stroke unit networks are well-developed in many European countries (13–19) but there is a lack of consistency in the application of treatment guidelines (20).

The provision of multidisciplinary, coordinated, structured rehabilitation, and appropriate specialist post-stroke health services—not only immediately after discharge from the stroke unit but also for months and years afterward—is critical for minimizing the long-term sequelae of stroke (21). This study aimed to deliver current information on high-quality long-term services for reducing stroke burden based on systematic reviews, guidelines, and primary literature on stroke care to optimize long-term post-stroke care.

## Rehabilitation settings and patient eligibility

**Figure 2:**
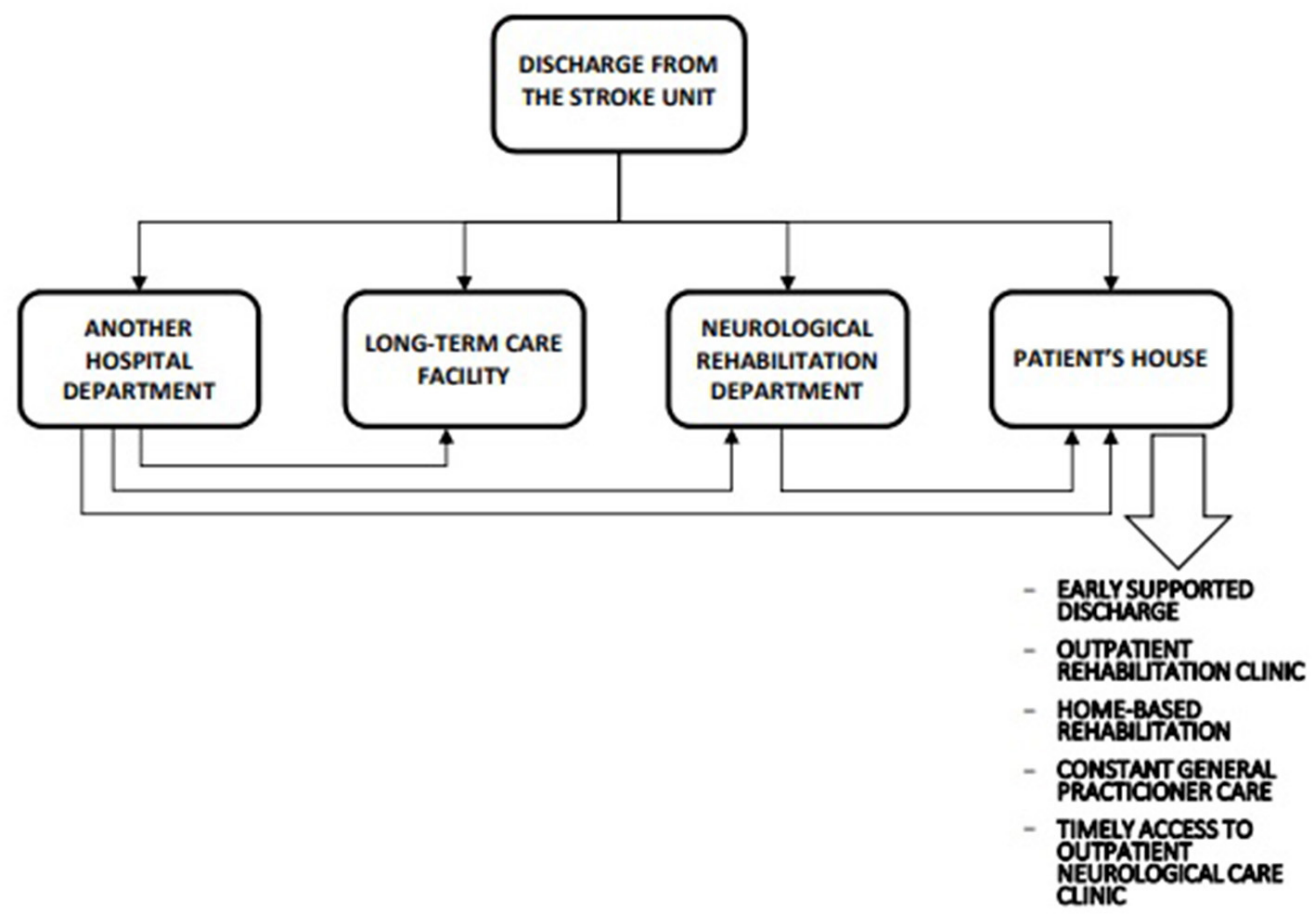
Optimal postacute stroke care pathway (unpublished - drawn by Jarosław Gębski, Polish Society of Health Economics, Warsaw, Poland).

Following a stroke, all survivors need care, support, and education; however, formal rehabilitation is only needed by patients with neurologic deficits affecting their functions. Although 20% of survivors of stroke (or over 30% of those treated with intravenous thrombolysis) fully recover by 2 weeks post-stroke (22), another 20% of them have severe functional deficits and require lifelong assistance with basic activities of daily living (ADL) despite rehabilitation (23, 24), and the remaining survivors have varying degrees of disability and need specific post-stroke rehabilitation (24). An optimal post-acute stroke care pathway to manage these patients is outlined in Figure 2.

In determining the most appropriate form of rehabilitation after discharge from the stroke unit, it is important to take into account the patient's general medical condition, neurologic findings, degree of disability (evaluated using standardized tests), mental and psychological statuses, and ability to participate in a rehabilitation program as well as the availability of caregiver support. Rehabilitation needs should be evaluated by a clinician experienced in neurologic assessment or by a multidisciplinary team as soon as the patient's medical and neurologic condition permits (24–26) to determine the appropriate intensity of rehabilitation and allocation of relevant resources.

Criteria for a comprehensive in-hospital rehabilitation program include a stable general condition, the ability to learn, sufficient physical endurance to sit unsupported for at least 1 h, and the ability to actively participate in rehabilitation (24). Initiation of such a rehabilitation program should be reserved for patients who have more than one type of disability and require the services of 2 or more rehabilitation disciplines (e.g., physiotherapy, occupational therapy, speech therapy, and neuropsychological therapy) (27). Patients with moderate disabilities and sufficient physical endurance to tolerate intensive rehabilitation (often at least 3 h per day of physically demanding activities) are the best candidates for such a program.

The decision of whether to admit patients who experienced a severe stroke to an in-hospital rehabilitation program is not straightforward. Severe stroke is defined as unconsciousness at the onset with severe unilateral or bilateral paresis (28, 29) or an early Functional Independence Measure (FIM) score of < 40 (30). Stroke severity may also be influenced by medical comorbidities that impact overall disability and make rehabilitation more challenging. Patients who experienced a severe stroke are less likely to achieve functional independence even over the long term (31); in these patients, younger age and the presence of a caregiver (32, 33) determine the extent of functional improvement with rehabilitation, although the provision of multidisciplinary stroke care in a highly specialized facility over an extended period can achieve significant results, to the extent that some will not require long-term care in a nursing facility and can be discharged with strong support from their family and the community (24). For severe stroke patients who are unable to participate in or are contraindicated for intensive multidisciplinary rehabilitation, appropriate care and rehabilitation should be provided in long-term care facilities (24).

Patients who experienced a mild stroke (early FIM score >80) can undergo rehabilitation at outpatient facilities, which potentially allows them to be more involved in self-care and take greater responsibility for their recovery. Outpatient stroke rehabilitation can be divided into early supported discharge (ESD), hospital-based outpatient rehabilitation, and community-based rehabilitation (34). ESD arose from the recognition that many survivors of stroke prefer being at home following a stroke and was developed to reduce the length of hospital stay and provide multidisciplinary rehabilitation in a patient's own home. Members of the ESD team should have specialized stroke care knowledge and should include a physiotherapist, an occupational therapist, and a nurse. A coordinator facilitates weekly meetings and assigns therapists to each patient (35). This approach to rehabilitation has been shown to reduce the duration of hospitalization in the stroke unit and the number of patients requiring institutional care following discharge and increase patients' independence in ADL at 6 months (36, 37).

The condition of patients who experienced a stroke may deteriorate after they are discharged from the hospital, resulting in a loss of independence in ADL and necessitating long-term institutional care (38). Outpatient therapy should be initiated following discharge from in-hospital stroke units as a continuation of therapy and may include hospital-based “day,” hospital programs, or home-based rehabilitation consisting of occupational therapy without or with physiotherapy (39).

Stroke rehabilitation requires long-term commitment (for at least 3–5 years after the stroke) (34); patients in the chronic phase (>6 months after the stroke) should have access to rehabilitation to prevent secondary complications resulting from immobilization and maintain a functional state (40, 41). Rehabilitation has many benefits even if it is not initiated early on, as functional improvements post-stroke can continue for a long period (20) although the patient's rehabilitation needs will evolve. For chronic stroke, the most effective mode of delivery of physiotherapy/occupational therapy is through a community rehabilitation program—which is usually home-based (42) or a self-management program—carried out under the periodic supervision and instruction of a therapist (20). An important factor limiting the provision of proper and continuous post-stroke rehabilitation is the insufficient number of rehabilitation professionals and nursing staff with specialist knowledge in the field of stroke.

## Spasticity management

Spasticity management is important for helping patients adhere to their care plan and setting realistic expectations regarding post-stroke rehabilitation. Spasticity, a complex movement disorder, is a common post-stroke complication caused by excessive muscle tone and stretch reflex resulting in clonus and spasms (43, 44) that contributes to functional impairment and reduces patients' ADL and quality of life (45, 46). The prevalence of post-stroke spasticity ranges from 19 to 92%; the timing of onset varies (44, 47, 48) and typically emerges between 1 and 6 weeks after the stroke (49). The anatomic pattern and severity of spasticity depend on the neurologic deficit, age at stroke onset, and lesion location and size. The heterogeneity of the manifestations of spasticity makes the rehabilitation process highly challenging. A standardized approach is needed to ensure that patients with post-stroke spasticity are diagnosed in a timely manner and receive care soon after its onset (50, 51). Patients with spasticity also need to be informed about their condition and the available treatments. Acute stroke teams often overlook early signs of spasticity, although early recognition of the symptoms could lead to receiving earlier treatment, achieving better outcomes, and avoiding long-term complications (52, 53). The post-stroke checklist was developed as an easy-to-use tool to identify and facilitate the proper treatment of long-term complications of a stroke, including spasticity (54). Patients with weakness or problems with limb dexterity, especially of the upper limb, that interfere with ADL and increase muscle stiffness in at least 1 joint at 4–12 weeks post-stroke are at high risk of developing severe spasticity and should be directly referred to a specialist who can administer botulinum toxin treatment and perform physiotherapy assessment (53).

## Secondary stroke prevention and management of early complications

Patients who experienced a chronic stroke have better outcomes when they receive effective treatment within an integrated care system with regular follow-up and self-management support (55, 56). They often receive complex information about risk factors for stroke recurrence, secondary prevention methods, treatment of comorbidities, lifestyle changes, and rehabilitation strategies at the time of discharge from the hospital. Providing this information can allow patients (and their families) to better care for their illnesses. The self-management model of care is essential for improving outcomes; therefore, stroke teams must support stroke survivors in transitioning to this care model (56–58).

General practitioners (GPs) play an integral role in the management of post-stroke patients. From the hospital, a GP should receive all the necessary information about the patient for secondary prevention and proper monitoring of medication use and lifestyle modifications in the primary care settings. In routine practice, the GP can identify deterioration in a patient's functioning post-discharge and arrange a referral for further therapy (59). The GP's involvement in stroke survivors' care alleviates their dependence (as well as that of their caregivers) on specialists and allows patients to better understand and manage their condition.

Another important element of post-stroke care is timely access to outpatient specialist neurologic care clinics linked to hospital services and primary care, with an initial visit at 6 months post-stroke and then one time a year as a long-term follow-up. The purpose of these visits is to monitor the patient's neurologic status and assess the occurrence and treatment of complications such as post-stroke cognitive disorders, depression, or epilepsy (29, 56, 59).

## Summary and conclusion

Experts have long suggested organizational solutions and goals of proper care for patients who experienced a stroke (20, 60, 61). Based on these recommendations, many countries are systematically improving the quality of acute stroke care, including the creation of better-functioning stroke unit networks. Current healthcare policy trends in many countries point to broader implementation of intravenous thrombolysis and mechanical thrombectomy, which are consistent with the ischemic stroke treatment guidelines (62). The proportion of patients receiving specific therapy for ischemic stroke is increasing, with successful outcomes in many cases. However, although doctors caring for patients in the acute phase are constantly improving their qualifications and acquiring highly specialized knowledge to implement acute stroke interventions properly and safely, they lack opportunities and time to develop competencies in neurorehabilitation and long-term post-stroke care.

Significant improvements in patient outcomes and healthcare savings may be afforded by improved access to rehabilitation and specialist outpatient neurologic care and their integration with primary care. Effective long-term post-stroke care requires optimal pathways and facilities for patients in different clinical conditions. The efficient organization of all hospital and community practice settings for post-stroke patients requires investment in infrastructure and continuous training of all healthcare professionals to ensure adequate provision of care. Decisions based on the principle of the effectiveness of continuous stroke care will ensure the most beneficial allocation of limited financial resources. As most post-stroke patients spend most of their lives outside of formal healthcare settings, it is essential to expand and coordinate partnerships with government sectors (e.g., the Ministry of Work and Social Policy), the private healthcare sector, non-governmental organizations, and community groups.

The optimal model of post-stroke care is not widely used because of a lack of coordination of such care and the dearth of medical professionals who can provide highly specialized post-stroke rehabilitation and long-term care. This study summarized the frequently overlooked problems in post-stroke patient care and outlined the necessary steps to organize a network of post-stroke care centers (both inpatient and outpatient) in individual countries with the active involvement of primary care physicians. A limitation of the proposed pathway is that it was developed based on the experience of experts and not on data from studies evaluating the effectiveness of such an approach; despite a general acknowledgment of the need for better organization of long-term stroke care, there is insufficient research and evidence to support experts' recommendations.

Currently, in many countries, patients who have had a stroke are monitored indirectly based on population health statistics. Some countries maintain stroke registries or conduct observational studies to monitor patients in the year after a stroke (21), but most registries focus solely on the quality of early stroke care. The creation of a register—for example, as an extension of the Registry of Stroke Care Quality that is organized based on the Stroke Action Plan for Europe—for long-term assessment of the quality of post-stroke care would allow for an easier analysis of the effectiveness of the proposed care scheme.

## Data availability statement

The original contributions presented in the study are included in the article/supplementary material, further inquiries can be directed to the corresponding author.

## Author contributions

IS-D contributed to the conception and design of the article, collected and organized the data, wrote the first draft of the manuscript, and revised, edited, and approved the final submitted version of the manuscript.
